# Prevalence of *Listeria Monocytogenes* and *Salmonella* spp. in Different Ready to Eat Foods from Large Retailers and Canteens over a 2-Year Period in Northern Italy

**DOI:** 10.3390/ijerph182010568

**Published:** 2021-10-09

**Authors:** Marta Castrica, Egon Andoni, India Intraina, Giulio Curone, Emma Copelotti, Francesca Romana Massacci, Valentina Terio, Silvia Colombo, Claudia Maria Balzaretti

**Affiliations:** 1 Department of Health, Animal Science and Food Safety “Carlo Cantoni”, University of Milan, Via dell’ Università 6, 26900 Lodi, Italy; India.intraina@unimi.it (I.I.); Emma.copelotti@studenti.unimi.it (E.C.); claudia.balzaretti@unimi.it (C.M.B.); 2 Department of Public Health, Agricultural University of Tirana, Rr “Pajsi Vodica” Koder-Kamez, 1023 Tirana, Albania; eandoni@ubt.edu.al; 3 Department of Veterinary Medicine, University of Milan, Via dell’Università 6, 26900 Lodi, Italy; giulio.curone@unimi.it; 4 Istituto Zooprofilattico Sperimentale dell’Umbria e delle Marche “Togo Rosati”, Via Gaetano Salvemini 1, 06126 Perugia, Italy; fr.massacci@izsum.it; 5 Department of Veterinary Medicine, University of Bari, Provincial Road to Casamassima Km 3, 70010 Valenzano, Italy; valentina.terio@uniba.it; 6 Chemservice S.r.l.-Lab Analysis Group, Via F. lli Beltrami, 15, Novate Milanese, 20026 Milan, Italy; silvia.colombo@chemservice.it

**Keywords:** RTE food, foodborne, food safety, *Listeria monocytogenes*, *Salmonella* spp., risk analysis

## Abstract

This study aims to give an overview of the prevalence of *Listeria monocytogenes* and *Salmonella* spp. in 9727 samples (2996 for *L. monocytogenes* and 6731 for *Salmonella* spp.) from different categories of ready-to-eat (RTE) foods, collected over 2 years from 28 large retailers and 148 canteens in the regions of northern Italy. The RTE samples were classified into two groups according to the preparation methods: (*i*) multi-ingredient preparations consisting of fully cooked food ready for immediate consumption, or with minimal further handling before consumption (Group A), and (*ii*) multi-ingredient preparations consisting of cooked and uncooked food, or preparations consisting of only raw ingredients (Group B). *L. monocytogenes* and *Salmonella* spp. were investigated in both of these categories. The overall prevalence of *L. monocytogenes* and *Salmonella* spp. was 0.13% and 0.07%, respectively. More specifically, *L. monocytogenes* was found in 0.04% of 2442 analysed RTE food samples belonging to group A and in 0.54% of 554 samples belonging to group B. Furthermore, 0.03% of 5367 RTE food samples from group A and 0.21% of 1364 samples from group B tested positive for *Salmonella* spp. In conclusion, the results obtained in this study can provide a significant contribution to *L. monocytogenes* and *Salmonella* spp. risk analysis in RTE foods.

## 1. Introduction

As a result of changes in lifestyles, the current economic systems, a curiosity for culinary dishes that are diverse and distant from our traditions [1], and, more recently, the effects of the COVID-19 pandemic [2], there has been an increase in the consumption of so-called ready-to-eat foods (RTE). In this regard, in Europe in 2021, the average volume of consumption of RTE meals per person, thus far, amounts to 15 kg. In fact, Italy exemplifies a growing trend whereby, in 2019, the consumption of these products was 5.6 kg per capita, and this prediction is expected to reach 6 kg of ready meals per capita by 2023 [3]. A study conducted in Italy in 2020 by Nils-Gerrit Wunsch [4] showed that 29% of the population over the age of 35 frequently consumed ready-to-eat meals.

The rise in consumer demands for ready-to-eat products certainly has positive aspects for the retail and foodservice industry. On the other hand, there are emerging microbiological issues concerning food safety that need to be considered [5]. In general, depending on the preparation method, RTE foods may be more or less prone to microbiological risks [6]. More specifically, with regard to multi-ingredient preparations consisting of fully cooked foods ready for immediate consumption, or with minimal further handling prior to consumption, these foods are relatively exposed to microbial risks because the cooking process and fast preparation period limit the possibility of and exposure to microbial risks. Alternatively, multi-ingredient RTE foods that are prepared solely using cooked and uncooked foods, or made using only raw ingredients (e.g., sandwiches and mixed salads), are potentially more exposed to microbial risks. The latter is due to the fact that during preparation, they are generally more manipulated by food business operators (FBOs) and, more significantly, they do not undergo any heat treatment (e.g., cooking) prior to consumption. Therefore, it could have a restorative effect on health compliance [7].

A factor that may affect the food safety of the finished product, especially for the latter RTE food category, is the microbiological profile (initial microbial load) of the ingredients that constitute the preparation. The latter occurs because the handling, processing, and storage phases can further aggravate a non-compliant or borderline starting situation [7]. For these reasons, the correct operating procedures by FBOs, from a hygiene and health point of view, are an indispensable prerequisite [8,9]. As reported in the literature, foodborne disease outbreaks related to RTE food are associated with various foodborne pathogens, including *L. monocytogenes* and *Salmonella* spp. [10,11]. *L. monocytogenes* is an intracellular, Gram-positive pathogenic bacterium [12]. It is ubiquitous and psychotropic [13], and these features make it a perfect candidate for the contamination of RTE foods. *Salmonella* spp., however, is a Gram-negative, rod-shaped bacteria belonging to the *Enterobacteriaceae* family that grows at 37 °C [14]. Members of *Salmonella* spp. are classified into serotypes, and infection in humans can occur through the ingestion of contaminated water or food. Generally, poultry, meat, and eggs are the principal vehicles for these foodborne pathogens [15]. Among illnesses from bacterial foodborne pathogens, salmonellosis and listeriosis are the leading causes of death related to bacterial foodborne infections [16]. In 2019, 2621 cases of listeriosis were reported, and the notification rates increased rapidly by age in the older age groups (over 65 years). This trend in Europe has confirmed that *L. monocytogenes* is the most serious zoonotic disease, with high rates of hospitalisation (92%) and mortality (17.6%) [17]. In particular, as described by Vázquez-Boland et al. [18], the risk and severity of listeriosis are significantly higher among the elderly, pregnant women, infants, and individuals with a compromised immune system, with an associated fatality rate of 16–25% despite treatment [19].

Regarding foodborne outbreaks by *Salmonella* spp., EFSA’s annual report [20] highlights that *Salmonella* spp. is the second most commonly reported gastrointestinal infection in humans after campylobacteriosis. In 2019, a total of 87,923 confirmed cases of salmonellosis in humans were reported within the EU [20]. Again, the population groups most affected were the elderly, pregnant women, and immunocompromised individuals [20]. Furthermore, *Salmonella* infection is more complicated: not only can the bacteria be acquired through many types of foods, but it can also be acquired from non-food sources (e.g., from direct contact between individuals and infected animals, including pets) [21,22]. For these reasons, and especially for the protection of the consumer and public health, quantitative risk assessment, control, and prevention are essential throughout the food chain. Adherence to good hygiene practices during food handling, storage, and distribution is also essential.

In this regard, the purpose of the present study is to give an overview of the prevalence of *L. monocytogenes* and *Salmonella* spp. Studied over a 2-year period in northern Italy in two RTE food categories: large retailers and canteens. The results obtained from the study can contribute, at both national and international levels, to the updating of figures concerning the assessment of the risk of *L. monocytogenes* and *Salmonella* spp. in RTE foods.

## 2. Materials and Methods

All RTE foods were collected over a 2-year period, specifically from July 2019 to March 2021. The samples were analysed at the Food Inspection Laboratory of the Dept of Health, Animal Science and Food Safety “Carlo Cantoni” (University of Milan). The Laboratory performs its procedures according to UNI EN ISO/IEC 17025:2018 quality standards (general requirements for the competence of testing and calibration laboratories).

### 2.1. Collection and Description of RTE Food Samples

A total of 9727 RTE food samples (2996 for *L. monocytogenes* and 6731 for *Salmonella* spp.) were collected every month from 28 large retailers and 148 canteens in northern Italy.

A total of 250g of each RTE food sample was collected and placed in sterile plastic bags. Immediately after the collection, all of the RTE samples were transported to the laboratory in isothermal containers with ice (4 °C ± 2) and were analysed on the same day. In this study, RTE foods were divided into two groups according to their preparation methods and according to the categorisation standards provided by Ce.IRSA [23] (Table 1). 

### 2.2. RTE Sample Analysis

The microbiological analyses were focused on the detection of pathogenic microorganism markers, specifically *L. monocytogenes* and *Salmonella* spp. To carry out the analyses, 25 g of the food samples was homogenised using a Stomacher 400 (Stomacher 400 Circulator, Seward Ltd., Norfolk, UK) for 60 s, in 225 mL of appropriate enrichment broths.

For the *L. monocytogenes*, a volume of 0.1 mL of the initial food suspension (25 g of samples enriched with 225 mL of Listeria ½ Fraser Broth) was briefly plated in a selective chromogenic medium: rapid *L. mono*. The final test for the confirmation of suspected colonies was carried out by biochemical testing (Microgen™ Listeria-ID, Camberley, England), whereas, for the testing of *Salmonella* spp., a 25 g sample was pre-enriched and homogenised in 225 mL of buffered peptone water. Then, following the predetermined incubation time (Table 2), 0.1 mL of the pre-enriched sample was transferred to 10 mL of RVS broth (enrichment phase) and 1 mL of MKTTn broth (enrichment phase). Finally, after its incubation at 41.5 °C ± 1 °C for 24 h ± 3 h and 37 °C ± 1 °C for 24 h ± 3 h, respectively, 10 µL of the RVS broth was transferred to an XLD agar plate, and the same procedure was repeated by also plating the RVS broth onto a BGA agar plate. Equally, 10 µL of the MKTTn broth was plated in a BGA and XLD agar plate. After the fixed incubation period (Table 2), the confirmation of presumptive positive results was performed by biochemical testing (API 20 E NE) and by using Poly A-S + Vi and Poly H antisera. For both pathogens, the results are expressed as absence/presence in 25 g of the sample material.

In Table 2, the incubation times and temperatures are shown alongside the reference methods in relation to the specific pathogens and manufacturers of consumables.

### 2.3. Statistical Analysis

All of the results were recorded using Microsoft Excel 2010 software (Microsoft, Redmond, WA, USA). The prevalence and confidence intervals were calculated using Statistical Package R software, and the statistical significance among the RTE food categories was investigated using Pearson’s chi-squared test. An AP value of 0.05 was considered as statistically significant.

## 3. Results and Discussion

A high level of public health protection is one of the fundamental objectives of the food laws established in accordance with Regulation (EC) No. 178/2002. In accordance with Regulation (EC) No. 2073/2005 on microbiological criteria, the acceptability criteria are defined in relation to the different microorganisms of a food product.

Specifically, the limits set for the presence of *L. monocytogenes* in food products are the absence of the pathogen in 25 g of the sample or 100 CFU/g in RTE foods which are able of supporting the growth of the micro-organism. Whereas the limits set for *Salmonella* spp. are the absence of the bacteria in 25 g of raw food and its absence in 10g of cooked food.

**Figure 1:**
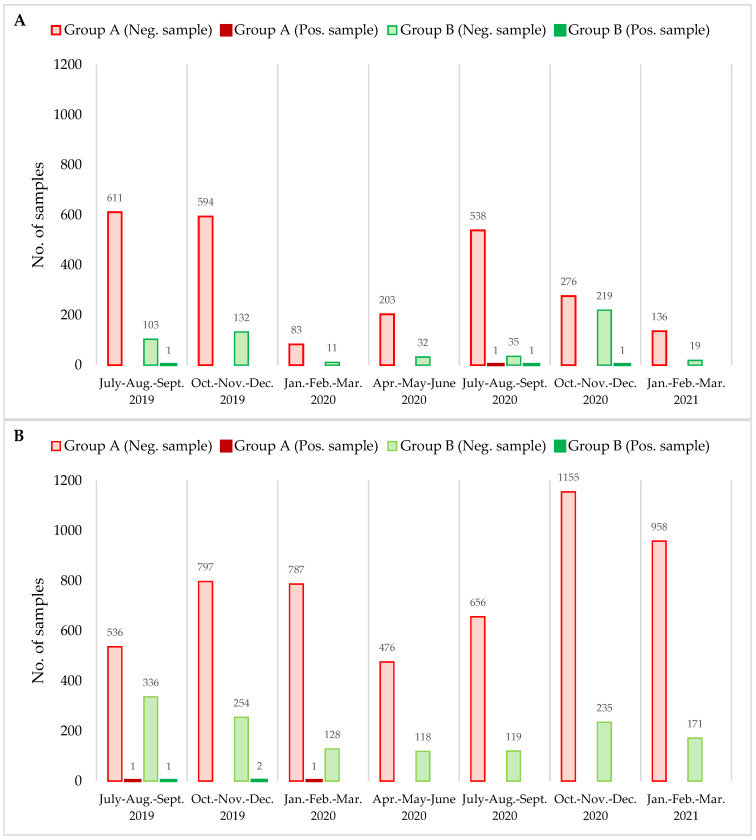
Summary of results in the different ready-to-eat food groups split into trimesters: (**A**) *Listeria monocytogenes* and (**B**) *Salmonella* spp.

All of the RTE samples analysed in this study were evaluated on the basis of the criteria proposed in the above-mentioned European Regulation. Table 3 and Table 4 show the results of the analysis of the prevalence of *L. monocytogenes* (LM) and *Salmonella* spp. (S spp.) in two groups (A and B) of RTE food samples. The monitoring was performed from July 2019 to March 2021 and highlighted that, out of a total of 2.996 samples tested for the absence/presence of LM, four samples were positive (0.13%, Figure 1A), while for S spp., five samples tested positive out of a total of 6731 (0.07%, Figure 1B). In group A, 1 out of 2442 (0.04%) samples tested positive for LM while, in group B, 3 out of 554 (0.54%) tested positive for the bacterium. The number of positive samples for LM was found to be different between groups (*p* = 0.02). In group A, LM was found in a pasta sample, while in group B it was found in a salmon poke, ham, and fish salad. These findings suggest that multi-ingredient preparations of RTE meals composed of cooked and uncooked food, or preparation consisting of only raw ingredients, are more exposed to the risk of microbial contamination compared to multi-ingredient preparations. The latter, in fact, fully composed of only cooked food, ready to eat or with minimum further handling before consumption. For this reason, the adoption and implementation of good hygiene practices by operators handling ingredients is of fundamental importance to minimise the risk of bacterial contamination [7]. Although it is not possible to directly compare data from other studies [24] due to the many variables that can influence the analysis, such as the geographical area and the observation period, it is interesting to note that the official data reported at a national level confirm our results [25]. Indeed, the Italian Ministry of Health, in its review of the assessment conducted in January 2021 of the risk of the consumer’s exposure to *L. monocytogenes* [25], showed contamination rates for raw fish and seafood products of 0.6%, while cured meat products, such as cooked ham and cured ham (2%), were among the foods with the highest positive rates. Compared with several studies in other countries, the data regarding *L. monocytogenes* isolates recovered from the RTE food products are varied. Gormley et al. [26] reported the prevalence of *L. monocytogenes* in foods from markets and specialty food shops in the UK that were very similar to those in northern Italy. Specifically, out of a total of 2359 samples analysed, 2352 (99.7%) samples were satisfactory and six samples (0.3%) were unsatisfactory. It is important, however, to highlight that the sampling focused on a specific category of ready-to-eat foods: specialty meats.

In contrast, Koskar et al. [24] analysed the prevalence of *L. monocytogenes* in a total of 30.016 RTE foods, which shows very different results to those attained in our study. Indeed, 3.6% of the samples were found to be positive, and the highest prevalence of bacteria was found in RTE fish and fish products (11.6%). These higher results, compared to ours, may also be influenced by a bigger sample size (2442 vs. 30,106).

The obtained results for *Salmonella* spp. (Table 4 and Figure 1B) demonstrate that the overall positive results in a total of 6.731 samples is 0.07%, which corresponds to five positive samples. In this case, two positive samples (lasagne and roast pork) were found in group A out of a total of 5.367 samples analysed (0.03%). In the analysed samples belonging to group B, the presence of S spp. was detected in three samples (salami and two chicken salads) out of 1.364, equal to 0.21%. The chi-squared test did not show differences between the groups (*p* > 0.05).

Again, our results appear to be significantly lower than those found in other works. In particular, Yang et al. [27], over 3 years of sampling and a total of 539 RTE food samples, analysed 19 (3.5%) that were positive for *Salmonella*, including 3 (2.6%) of 117 cooked pork samples; 3 (2.0%) of 152 cooked chicken samples; 8 (6.6%) of 121 cooked duck samples; 2 (3.7%) of 54 cold vegetable dishes in sauce samples; 2 (3.9%) of 51 cold noodles in sauce samples; and 1 (2.3%) of 44 fried rice/sushi samples. Although no similar prevalence was found, some categories of RTE food that were found positive for *Salmonella* by Yang et al. [27] were also found positive in our study, such as pork and chicken RTE products. As already highlighted, this disparity can be attributed to different factors, such as the different geographic locations of sampling, the sample size, and the sampling period. Indeed, the work of Kramarenko et al. [28] showed results more in line with ours. Specifically, concerning a total of 264 RTE foods, the overall prevalence for *Salmonella* in non-thermally processed food was 0.81% (256/31,576) and in RTE products only 0.02% (4/16,351).

In general, it is important to highlight that the reported incidence in different countries for *L. monocytogenes* and *Salmonella* spp. varies greatly [29,30]. In fact, Kurpas et al. [29] showed that the prevalence of *L. monocytogenes* investigated within the same sample type (RTE meat products) in different countries (EU and extra EU) has a wide range, from 0.24% (UK) to 58.3% (Slovakia). At the same time, Effimia et al. [30] also showed differences in the incidence of *Salmonella* spp. within the same country but in different geographical locations, again, with widely varying results (0.9% in Kefalonia vs. 12.5% in northern Greece).

In addition, Effimia et al. [30] reported results very close to ours. In fact, for meat cooked with vegetable meals, from a sample in restaurants in Kefalonia (Greece), the incidence of *L. monocytogenes* showed a range from 0.0% to 2.4%, while *Salmonella* spp. was not detected in cooked meat with vegetables, vegetables with olive oil (salads), or ice cream.

In relation to our study, these differences in prevalence show that other factors, besides those described above (i.e., sample size, sampling period, and geographic location), may have an impact on the results, namely how the large retailers and/or canteens apply their own good hygiene practice procedures during the preparation, handling, and storage of RTE foods.

The incidence of the potential foodborne pathogens LM and S spp. in the samples of this study was low. Nevertheless, it is essential to highlight that RTE foods, if subjected to inappropriate handling practices and abuse of time and temperature during processing and distribution, can represent a favourable medium for the development of pathogenic microorganisms in humans. Indeed, as reported by several authors [31,32], *L. monocytogenes* and *Salmonella* spp. are responsible for 97% of foodborne diseases associated with catering systems. 

Furthermore, with the rise in better practices for surplus food recovery, RTE food from catering events or collective catering is frequently recovered by charitable organisations and redistributed to people who experience food insecurity. As reported by Gowda et al. [33], very often, food insecurity may lead to poor health, thus expanding the population’s susceptibility to foodborne diseases. For this reason, it is of paramount importance that all actors in the food supply chain, from the donor companies to the charitable organisations (recipient organisations), apply good hygienic practices (GHP) and good manufacturing practices (GMP) [34].

The exposure of highly susceptible people to contaminated food may contribute to the burden of disease [17], but this can be prevented by improving the risk analysis at different stages of the food chain. Moreover, maintaining high standards of hygiene through an environmental monitoring program should be adopted, with the aim of minimising the risks of cross-contamination (e.g., between raw products and RTE foods). Training activities should be intensified, since workers are frequently the means by which the transmission of pathogens occurs, and a proper compliance to temperature is fundamental for the safety of RTE foods [8,35]. As reported by Kotzekidou [5], the safety of RTE foods depends on the use of appropriate raw ingredients; the processing operation; and other parameters intrinsic to the ingredients, such as pH and water activity (aw).

For these reasons, the application of new technologies on fresh food items, such as active or smart packaging [36] and rapid screening methods (e.g., molecular tests) [37,38], can be useful and innovative tools for foodborne disease control and surveillance.

Moreover, low-temperature storage, to limit the growth of foodborne pathogens, as well as heat treatment, can positively affect the safety of foods. Temperature control and maintenance are essential to reduce the growth and survival rates of foodborne pathogens in RTE foods. Furthermore, another control point is the cleanliness of food contact surfaces [39], food preparation surfaces, and utensils, as they may be a *reservoir* for microbial contamination, and, as reported by Djordjevic et al. [40], *L. monocytogenes* can form biofilms on food-processing surfaces, potentially leading to food product contamination. It is, therefore, necessary to reiterate the importance of proper handling, surface cleaning, and storage, both at the distribution administration stages and at home. It is necessary to communicate to consumers that it is important to avoid the long storage of ready-to-eat products purchased in large and small retailers.

Several studies have confirmed that food handler training can be effective for the improvement of food service systems and also for the identification of deficient practices [41,42,43]. Finally, prerequisite programs and HACCP are essential in food production environments with a high risk of *L. monocytogenes* and *Salmonella* spp. to minimise the risks of contamination of food products. 

Another important aspect to be explored in a future study involves changes to food production practices and the evolution of foodborne pathogens that generates a new public health concern, such as antimicrobial resistance. Antibiotic resistance among foodborne microorganisms is an ongoing public health threat that continues to be a challenge [44]. Although efforts are necessary to limit the misuse of antibiotics, 33,000 deaths per year have been estimated in Europe [45]. It will be a significant task to characterise the antibiotic-resistant foodborne pathogens, the factors that have contributed to their emergence, their antibiotic resistance mechanisms, the public health implications of their spread through the food supply chain, and potential antibiotic alternatives for their control, from a “One Health” perspective.

## 4. Conclusions

The aim of the study is to show the monitoring, over a period of about two years in northern Italy, of pathogenic microorganisms, such as *L. monocytogenes* and *Salmonella* spp., in RTE food collected in retailers and canteens. Although our results show a low prevalence of the two pathogens researched, which is positive in relation to the mandatory EU regulations requiring their absence, a control system in relation to production characteristics and supply chain flows is still essential for the food safety control of RTE foods. The procedures to be implemented are GMP, GHP, and Sanitation Standard Operating Procedures (SSOP) in primary production as well as creating increasingly integrated systems with standardised management procedures. In conclusion, the obtained results might contribute, at both the national and the international levels, to the updating of data on the risk assessment of *L. monocytogenes* and *Salmonella* spp. in RTE foods.

## Figures and Tables

**Table 1:** Ready-to-eat food groups involved in the study.

ID Groups	Description of Ready-to-Eat Groups	No. of Samples per Parameter		No. of Samples Per Sampling Site			
				*L. monocytogenes*		*Salmonella* spp.	
		*L. monocytogenes*	*Salmonella* spp.	Large retailers	Canteens	Large retailers	Canteens
Group A	Multi-ingredient preparations composed of fully cooked food, ready for immediate consumption or with minimum further handling before consumption (e.g., pasta, pizza, burgers, vegetables, ready meals after regeneration, whole pies, sausage rolls, quiches, roast meats, and chicken portions).	2442	5367	143	2299	167	5200
Group B	Multi-ingredient preparations composed of cooked and uncooked food or preparation consisting only of raw ingredients (e.g., seafood sauces, roast beef with raw rocket, mixed salads, julienned carrots, sliced fennel, chopped lettuce, radicchio, and pre-prepared fruit salads).	554	1364	228	326	361	1003

**Table 2:** Culture techniques (media and agar), incubation conditions, and the biochemical and serological confirmation tests used for the microbiological testing of ready-to-eat food samples.

Parameters	Culture Techniques	Incubation		Culture Media and Agar	Biochemical Confirmation Test	Serological Testing	Reference Method
		Time (h)	Temp. (°C)				
*L. monocytogenes* detection	Enrichment	24	30	Listeria ½ Fraser ^1^	Microgen™ Listeria-ID ^3^		AFNOR BRD 07/04-09/98
	Plate	24–48	37	RAPID’ *L. mono* ^2^			
*Salmonella* spp. detection	Pre-enrichment	18	37	buffered peptone water ^1^	API 20 E NE ^4^	Poly A-S + Vi ^5^ Poly H ^5^	ISO 6579:2017
	Selective enrichment	24	41.5	Rappaport-Vassiliadis broth (RVS) ^1^			
		24	37	Muller-Kauffmann Tetrathionate-Novobiocin broth (MKTTn) ^1^			
	Plate	24	37	Xylose lysine Desoxycholate Agar (XLD) ^1^			
		24	37	Brilliant Green Agar (BGA) ^1^			

^1^ Thermo Fisher, Waltham, USA. ^2^ Bio-Rad, Marne la Coquette, France. ^3^ Microgen Biproducts, Camberley, England. ^4^ bioMérieux, Marcy l’Étoile, France. ^5^ Biogenetics Diagnostics Srl, Padua, Italy.

**Table 3:** *Listeria monocytogenes* in ready-to-eat foods, from July 2019 to March 2021.

	No. Positive/Total No. of Samples (% Positive)								
ID Groups	July–Aug.–Sept. 2019	Oct.–Nov.–Dec. 2019	Jan.–Feb.–Mar. 2020	Apr.–May–June 2020	July–Aug.–Sept. 2020	Oct.–Nov.–Dec. 2020	Jan.–Feb.–Mar. 2021	Total	CI_95_ of % positive ^1^
Group A	0/611 (0)	0/594 (0)	0/83 (0)	0/203 (0)	1 ^a^/539 (0.18)	0/276 (0)	0/136 (0)	1/2442 (0.04)	0.01–0.25
Group B	1 ^b^/104 (0.96)	0/132 (0)	0/11 (0)	0/32 (0)	1 ^c^/36 (2.77)	1 ^d^/220 (0.45)	0/19 (0)	3/554 (0.54)	0.18–1.58
Total	1/715 (0.13)	0/726 (0)	0/94 (0)	0/235 (0)	2/575 (0.34)	1/496 (0.20)	0/155 (0)	4/2996 (0.13)	
CI_95_ (%) ^1^	0.02–0.79	0.00–0.53	0.00–3.93	0.00–1.61	0.10–1.26	0.04–1.13	0.00–2.42	0.05–0.34	

^1^ CI_95_: 95% confidence interval. Positive sample type: ^a^ pasta, ^b^ salmon poke, ^c^ fish salad, and ^d^ ham.

**Table 4:** *Salmonella* spp. in ready-to-eat food, from July 2019 to March 2021.

	No. Positive/Total No. of Samples (% Positive)								
ID Groups	July–Aug.–Sept. 2019	Oct.–Nov.– Dec. 2019	Jan.–Feb.– Mar. 2020	Apr.–May–June 2020	July–Aug.– Sept. 2020	Oct.–Nov.– Dec. 2020	Jan.–Feb.– Mar. 2021	Total	CI_95_ of % positive ^1^
Group A	1 ^a^/537 (0.18)	0/797 (0)	1 ^b^/788 (0.12)	0/476 (0)	0/656 (0)	0/1.155 (0)	0/958 (0)	2/5367 (0.03)	0.01–0.14
Group B	1 ^c^/337 (0.29)	2 ^d^/256 (0.78)	0/128 (0)	0/118 (0)	0/119 (0)	0/235 (0)	0/171 (0)	3/1364 (0.21)	0.07–0.64
Total	2/874 (0.22)	2/1053 (0.18)	1/916 (0.10)	0/594 (0)	0/775 (0)	0/1390 (0)	0/1129 (0)	5/6731 (0.07)	
CI_95_ (%) ^1^	0.06–0.83	0.05–0.69	0.02–0.62	0.00–0.64	0.00–0.49	0.00–0.28	0.00–0.34	0.03–0.17	

^1^ CI_95_: 95% confidence interval. Positive sample type: ^a^ lasagne, ^b^ roast pork, ^c^ chicken salad, and ^d^ salami and chicken salad.

## Data Availability

Not applicable.

## References

[1] Matera A., Altieri G., Ricciardi A., Zotta T., Condelli N., Galgano F., Genovese F., Di Renzo G.C. (2020). Microbiological stability and overall quality of ready-to-heat meals based on traditional recipes of the Basilicata region. Foods.

[2] James D., Bowness E., Robin T., McIntyre A., Dring C., Desmarais A., Wittman H. (2021). Dismantling and rebuilding the food system after COVID-19: Ten principles for redistribution and regeneration. J. Agric. Food Syst. Community Dev..

[3] STATISTA Ready-to-Eat Meals (Italy). https://www.statista.com/outlook/cmo/food/convenience-food/ready-to-eat-meals/italy.

[4] STATISTA Italy: Frequency of Ready Meals Consumption by Age 2020. https://www.statista.com/statistics/1182258/frequency-of-ready-meals-consumption-by-age-italy/.

[5] Kotzekidou P. (2013). Microbiological examination of ready-to-eat foods and ready-to-bake frozen pastries from university canteens. Food Microbiol..

[6] Redmond E.C., Griffith C.J., Slader J., Humphrey T.J. (2004). Microbiological and observational analysis of cross contamination risks during domestic food preparation. Br. Food J..

[7] Balzaretti C.M., Marzano M.A. (2013). Prevention of travel-related foodborne diseases: Microbiological risk assessment of food handlers and ready-to-eat foods in northern Italy airport restaurants. Food Control.

[8] Christison C.A., Lindsay D., von Holy A. (2008). Microbiological survey of ready-to-eat foods and associated preparation surfaces in retail delicatessens, Johannesburg, South Africa. Food Control.

[9] Beuchat L.R., Ryu J.H. (1997). Produce Handling and Processing Practices. Emerg. Infect. Dis..

[10] Gibbons I.S., Adesiyun A., Seepersadsingh N., Rahaman S. (2006). Investigation for possible source(s) of contamination of ready-to-eat meat products with Listeria spp. and other pathogens in a meat processing plant in Trinidad. Food Microbiol..

[11] Gilbreth S.E., Call J.E., Wallace F.M., Scott V.N., Chen Y., Luchansky J.B. (2005). Relatedness of Listeria monocytogenes isolates recovered from selected ready-to-eat foods and listeriosis patients in the United States. Appl. Environ. Microbiol..

[12] Farber J.M. (1991). Microbiological aspects of modified-atmosphere packaging technology—A review. J. Food Prot..

[13] Modzelewska-Kapituła M., Maj-Sobotka K. (2013). The microbial safety of ready-to-eat raw and cooked sausages in Poland: Listeria monocytogenes and Salmonella spp. occurrence. Food Control.

[14] D’Aoust J.Y., Maurer J., Doyle M.P., Beuchat L.R. (2007). Salmonella species. Food Microbiology. Fundamentals and Frontiers.

[15] Heredia N., García S. (2018). Animals as sources of food-borne pathogens: A review. Anim. Nutr..

[16] Burnett J., Wu S.T., den Bakker H.C., Cook P.W., Veenhuizen D.R., Hammons S.R., Singh M., Oliver H.F. (2020). Listeria monocytogenes is prevalent in retail produce environments but Salmonella enterica is rare. Food Control.

[17] Ricci A., Allende A., Bolton D., Chemaly M., Davies R., Fernández Escámez P.S., Girones R., Herman L., Koutsoumanis K., Nørrung B. (2018). Listeria monocytogenes contamination of ready-to-eat foods and the risk for human health in the EU. EFSA J..

[18] Vázquez-Boland J.A., Domínguez-Bernal G., González-Zorn B., Kreft J., Goebel W. (2001). Pathogenicity islands and virulence evolution in Listeria. Microbes Infect..

[19] Scharff R.L. (2012). Economic burden from health losses due to foodborne illness in the united states. J. Food Prot..

[20] EFSA—European Food Safety Authority (2019). The European Union One Health 2018 Zoonoses Report. EFSA J..

[21] Crim S.M., Griffin P.M., Tauxe R., Marder E.P., Gilliss D., Cronquist A.B., Cartter M., Tobin-D’Angelo M., Blythe D., Smith K. (2015). Preliminary incidence and trends of infection with pathogens transmitted commonly through food—Foodborne Diseases Active Surveillance Network, 10 U.S. sites, 2006–2014. MMWR. Morb. Mortal. Wkly. Rep..

[22] WHO—World Health Organization. https://www.who.int/news-room/fact-sheets/detail/salmonella-(non-typhoidal).

[23] CeIRSA—Centro Iterdipartimentale di Ricerca e Documentazione sulla Sicurezza Alimentare. https://www.ceirsa.org/matrice_alim.php#inizio.

[24] Koskar J., Kramarenko T., Meremäe K., Kuningas M., Sõgel J., Mäesaar M., Anton D., Lillenberg M., Roasto M. (2019). Prevalence and numbers of listeria monocytogenes in various ready-to-eat foods over a 5-year period in Estonia. J. Food Prot..

[25] Ministero della Salute Listeriosi Di Origine Alimentare: Valutazione Del Rischio Di Esposizione Per Il Consumatore. https://www.salute.gov.it/portale/documentazione/p6_2_2_1.jsp?lingua=italiano&id=3049.

[26] Gormley F.J., Little C.L., Grant K.A., de Pinna E., McLauchlin J. (2010). The microbiological safety of ready-to-eat specialty meats from markets and specialty food shops: A UK wide study with a focus on Salmonella and Listeria monocytogenes. Food Microbiol..

[27] Yang X., Huang J., Wu Q., Zhang J., Liu S., Guo W., Cai S., Yu S. (2016). Prevalence, antimicrobial resistance and genetic diversity of Salmonella isolated from retail ready-to-eat foods in China. Food Control.

[28] Kramarenko T., Nurmoja I., Kärssin A., Meremäe K., Hörman A., Roasto M. (2014). The prevalence and serovar diversity of Salmonella in various food products in Estonia. Food Control.

[29] Kurpas M., Wieczorek K., Osek J. (2018). Ready-to-eat meat products as a source of Listeria monocytogenes. J. Vet. Res..

[30] Effimia E. (2015). Prevalence of Listeria monocytogenes and Salmonella spp. in Ready-to-Eat Foods in Kefalonia, Greece. J. Bacteriol. Parasitol..

[31] Pérez-Rodríguez F., Valero A., Carrasco E., García R.M., Zurera G. (2008). Understanding and modelling bacterial transfer to foods: A review. Trends Food Sci. Technol..

[32] Cabedo L., Picart I Barrot L., Teixidó I Canelles A. (2008). Prevalence of Listeria monocytogenes and Salmonella in ready-to-eat food in Catalonia, Spain. J. Food Prot..

[33] Gowda C., Hadley C., Aiello A.E. (2012). The association between food insecurity and inflammation in the US adult population. Am. J. Public Health.

[34] Milicevic V., Colavita G., Castrica M., Ratti S., Baldi A., Balzaretti C.M. (2016). Risk assessment in the recovery of food for social solidarity purposes: Preliminary data. Ital. J. Food Saf..

[35] Oliveira N.A., Bittencourt G.M., Oliveira C.A.F. (2019). Listeria monocytogenes in Brazilian foods: Occurrence, risks to human health and their prevention. Curr. Res. Nutr. Food Sci..

[36] Dobrucka R., Cierpiszewski R. (2014). Active and Intelligent Packaging Food—Research and Development—A Review Renata Dobrucka *, Ryszard Cierpiszewski Department of Industrial Products Quality and Ecology, Faculty of Commodity Science. Pol. J. Food Nutr. Sci..

[37] Castrica M., Panseri S., Siletti E., Borgonovo F., Chiesa L., Balzaretti C.M. (2019). Evaluation of smart portable device for food diagnostics: A preliminary study on Cape Hake fillets (M. Capensis and M. Paradoxus). J. Chem..

[38] Castrica M., Chiesa L.M., Nobile M., De Battisti F., Siletti E., Pessina D., Panseri S., Balzaretti C.M. (2021). Rapid safety and quality control during fish shelf-life by using a portable device. J. Sci. Food Agric..

[39] Osimani A., Garofalo C., Clementi F., Tavoletti S., Aquilanti L. (2014). Bioluminescence ATP monitoring for the routine assessment of food contact surface cleanliness in a university canteen. Int. J. Environ. Res. Public Health.

[40] Djordjevic D., Wiedmann M., McLandsborough L.A. (2002). Microtiter plate assay for assessment of Listeria monocytogenes biofilm formation. Appl. Environ. Microbiol..

[41] Buccheri C., Mammina C., Giammanco S., Giammanco M., Guardia M.L., Casuccio A. (2010). Knowledge, attitudes and self-reported practices of food service staff in nursing homes and long-term care facilities. Food Control.

[42] Santana N.G., Almeida R.C.C., Ferreira J.S., Almeida P.F. (2009). Microbiological quality and safety of meals served to children and adoption of good manufacturing practices in public school catering in Brazil. Food Control.

[43] Veiros M.B., Proença R.P.C., Santos M.C.T., Kent-Smith L., Rocha A. (2009). Food safety practices in a Portuguese canteen. Food Control.

[44] EFSA (2020). The European Union Summary Report on Antimicrobial Resistance in zoonotic and indicator bacteria from humans, animals and food in 2017/2018. EFSA J..

[45] O’Neill J. (2016). Review on Antimicrobial Resistance: Tackling Drug-Resistant Infections Globally: Final Report and Recommendations.

